# Towards third generation matrix metalloproteinase inhibitors for cancer therapy

**DOI:** 10.1038/sj.bjc.6603043

**Published:** 2006-03-14

**Authors:** C M Overall, O Kleifeld

**Affiliations:** 1 CBCRA Program in Breast Cancer Metastasis, Departments of Oral Biological & Medical Sciences, Biochemistry & Molecular Biology, The UBC Centre for Blood Research, University of British Columbia, Vancouver, BC, Canada V6T 1Z3

**Keywords:** target validation, antiproteolytic drug, cancer therapy, drug design, zinc chelation

## Abstract

The failure of matrix metalloproteinase (MMP) inhibitor drug clinical trials in cancer was partly due to the inadvertent inhibition of MMP antitargets that counterbalanced the benefits of MMP target inhibition. We explore how MMP inhibitor drugs might be developed to achieve potent selectivity for validated MMP targets yet therapeutically spare MMP antitargets that are critical in host protection.

Twenty five years ago, the therapeutic strategy of controlling cancer by broadly targeting collagenase (matrix metalloproteinase (MMP)1), stromelysin-1 (MMP3), and gelatinase A (MMP2), the three then known MMPs, was founded on reducing degradation of basement membrane and extracellular matrix proteins by cancer cells in metastasis and angiogenesis (Liotta *et al*, 1980; Hodgson, 1995). In the development of MMP inhibitors (MMPI) as anticancer drugs, the first generation peptidomimetic compounds (batimastat, BB94; GM-6001 ilomostat) were not orally bioavailable. These were superseded by second generation MMPIs from several companies that entered phase III clinical trials to treat many types of advanced cancer (Zucker *et al*, 2000; Fingleton, 2003). However, the broad spectrum MMPIs as well as those that show partial selectivity failed in extensive phase III clinical trials (Zucker *et al*, 2000; Coussens *et al*, 2002; Overall and López-Otin, 2002; Fingleton, 2003). As a family, all 23 MMPs have been considered to be cancer drug targets, but is this correct? indeed none were properly validated until recently (reviewed by Overall and Kleifeld, 2006). So, can validated MMP drug targets in cancer be therapeutically blocked by highly selective third generation MMPIs to treat cancer?

Today, it is clear that the major role of MMPs is for homeostatic regulation of the extracellular environment and for controlling innate immunity (Overall, 2004; Parks *et al*, 2004), not simply to degrade extracellular matrix as their name suggests. In tumorigenesis, MMPs participate in many deregulated signaling pathways that are used by the tumour to promote cancer cell growth and angiogenesis, side-step apoptosis, and for evasion of protective host responses (McCawley and Matrisian, 2001; Egeblad and Werb, 2002). These sophisticated cellular control functions represent new avenues for the therapeutic control of cancer. Conversely, stromal cells harness the beneficial actions of MMPs in tissue homeostasis and innate immunity for host resistance against cancer (Overall and Kleifeld, 2006). All MMPs exhibit some of these functions, but MMPs -3, -8 and -9 have activities so important that when genetically knocked out, this leads to enhanced tumorigenesis and metastasis in some animal models of cancer (reviewed by Overall and Kleifeld, 2006). In drug development, antitargets are those molecules that must be therapeutically avoided to prevent worsening of disease or because of severe adverse side effects. A therapeutic opportunity for anticancer drugs occurs where blocking the detrimental activities of drug targets outweighs the loss of their beneficial actions. This opportunity was not attained by the second generation MMPIs, which failed to show clinical efficacy. Even worse, for patients taking the carboxylate MMPI BAY-12-9566, small cell lung cancer metastasis worsened. Musculoskeletal side effects also necessitated reduced dosing in some patients. If efficacy and adverse reactions occurred at similar doses, this raises concerns as to whether the minimal effective concentration was reached in all patients – no side effects might reflect noneffective drug concentrations. Coupled with clinical trial design and that the patient and disease stratification did not match the preclinical animal models, MMPIs inevitably failed to control advanced cancer (Zucker *et al*, 2000; Coussens *et al*, 2002; Overall and López-Otin, 2002; Fingleton, 2003). So, for successful cancer therapy based on MMP inhibition, the next generation of MMP inhibitor drugs must be selective against validated MMP targets but therapeutically spare MMP antitargets. With such selectivity, adverse reactions might also be minimised.

## MMP TARGETS AND ANTITARGETS IN CANCER

A validated drug target unambiguously contributes to the disease. A drug that reduces the activity of a target molecule and in so doing cures or results in improved patient outcome, is the best confirmation and validation of a target's importance in disease (Overall and Kleifeld, 2006). Other strong validation criteria comes from human genetic and genomic studies; very recent studies of MMP knockout mice crossed into transgenic oncogene-expressing backgrounds that spontaneously develop multistage cancer, and pathway validation using RNAi approaches *ex vivo*. Although the MMP family has long been suggested as promising targets in cancer, remarkably only three – MMP1, -2, -7 – we consider to have been experimentally validated sufficiently to be designated as cancer targets (reviewed in (Overall and Kleifeld, 2006)).

Reflecting the importance of MMPs in normal tissue homeostasis and host resistance in cancer, we also designated three other MMPs as validated antitargets (MMP3, -8, -9) (Overall and Kleifeld, 2006). In view of the well-documented pro-tumorigenic actions of MMP3 and -9 (Egeblad and Werb, 2002; Fingleton, 2003), this appears paradoxical. However, strong antitarget activities of a MMP will override any classification of it as a drug target, since the results of therapeutically blocking an antitarget are potentially so detrimental or even life threatening. These studies have been reviewed recently (Overall and Kleifeld, 2006). The pressures of drug development by the pharmaceutical industry coupled with the past negative clinical experiences of MMPIs means the criteria for MMP target classification will be of the strictest nature, as they should. Nonetheless, it is a harsh reality that may necessitate abandoning otherwise very promising MMP candidate drug targets. So with six MMPs categorised, the remaining 17 MMPs await the critical confirmatory experimental and human data for drug target classification in cancer. This is necessary so that the active site structures of all MMPs can be compared. Structure activity relationship (SAR) principles can then be used in the design of next generation MMPI drugs that discriminate between the MMP targets and antitargets in cancer.

## SUBSTRATE DEGRADOMICS ELUCIDATES *IN VIVO* PROTEASE FUNCTION

In target and antitarget validation, the pleiotropic roles of MMPs in cancer need to be understood. For this, the MMP substrate degradome (López-Otin and Overall, 2002) must be identified. Since biologically relevant substrates might differ from theoretical activities inferred from *in vitro* experiments – ‘*just because it can, does not mean it does*’ – the best method of substrate discovery is to identify protease-cleaved substrates in complex milieus (López-Otin and Overall, 2002; Tam *et al*, 2004). Similarly, detecting cleavage fragments of known substrates confirms proteolysis *in vivo* and might be useful as cancer biomarkers. Recognising this, proteomic approaches have been developed to rapidly identify new protease substrates (Bredemeyer *et al*, 2004; Overall *et al*, 2004; Tam *et al*, 2004; Butler and Overall, 2006).

Many new bioactive substrates and indirect effects on a plethora of signaling molecules and other proteases were proteomically identified in membrane type (MT)1-MMP-transfected MDA-MB-231 breast carcinoma cells (Tam *et al*, 2004). With our recent degradomic studies revealing similar results for other MMPs, richly diverse bioactive substrate degradomes appear to be a common feature of MMPs (Butler and Overall, 2006)(Dean and Overall unpublished data). Hence, the diverse pivotal roles of MMPs in tissue homeostasis and innate immunity are just beginning to be recognised.

## THE PROTEASE WEB

Many proteases and endogenous protease inhibitors are MMP substrates (reviewed in Overall, 2002), and recently many more have been identified such as secreted leukocyte protease inhibitor and cystatins (Tam *et al*, 2004; Butler and Overall, 2006; Dean and Overall unpublished data). This makes the development of MMPIs as anticancer drugs even more challenging, since like signalling pathways, proteolysis pathways do not function alone *in vivo.* Instead, they interact to form a dynamic web – the ‘protease web’ of interconnecting proteolytic systems, cascades and circuits (Overall and Kleifeld, 2006). Net activity of a protease, like the web, therefore depends on the activities of many proteases and inhibitors. In forging many cross-class and protease family connections, MMPs are some of the key nodal proteases of the protease web. So, by viewing proteolysis as a system, it is apparent that protease overexpression can lead to unexpected interactions that ripple across the protease web – much like an oscillating spider web on trapping an insect – that is gradually restored in a robust system. However, disruption of this balance can create an environment that promotes tumour growth and progression. A similar disruption of this web can occur when tissues are exposed to MMPI drugs (Butler and Overall, 2006), resulting in indirect off-target drug effects on unrelated proteases and their families, but all stemming from reduced MMP activity. These must be understood so that MMPIs can be designed to minimise perturbations in the protease web that manifest as side effects.

## TOWARD THIRD GENERATION MMPIS

In the face of selective pressures from the tissue-specific melieu at metastasis sites or from anticancer drugs, tumour cells phenotypically evolve, new MMP expression profiles emerge, and the antitarget substrates and subordinate pathways become less effective in host defense. By inhibiting multiple MMPs, broad-spectrum MMPIs are less likely to lead to resistance compared with more specific drugs. Despite this, it is now clear that successful MMPIs should ideally spare MMP antitargets by ∼3 log orders of difference in *K*_i_ over targets. Chemically, this is challenging due to similarities in MMP active sites. If this is not possible, then patient exposure might be reduced by shorter periods of dosing or alternative routes of administration. How then to move forward and design highly specific inhibitor drugs of validated MMP targets?

### S_1_′ specificity loop

**Figure 1:**
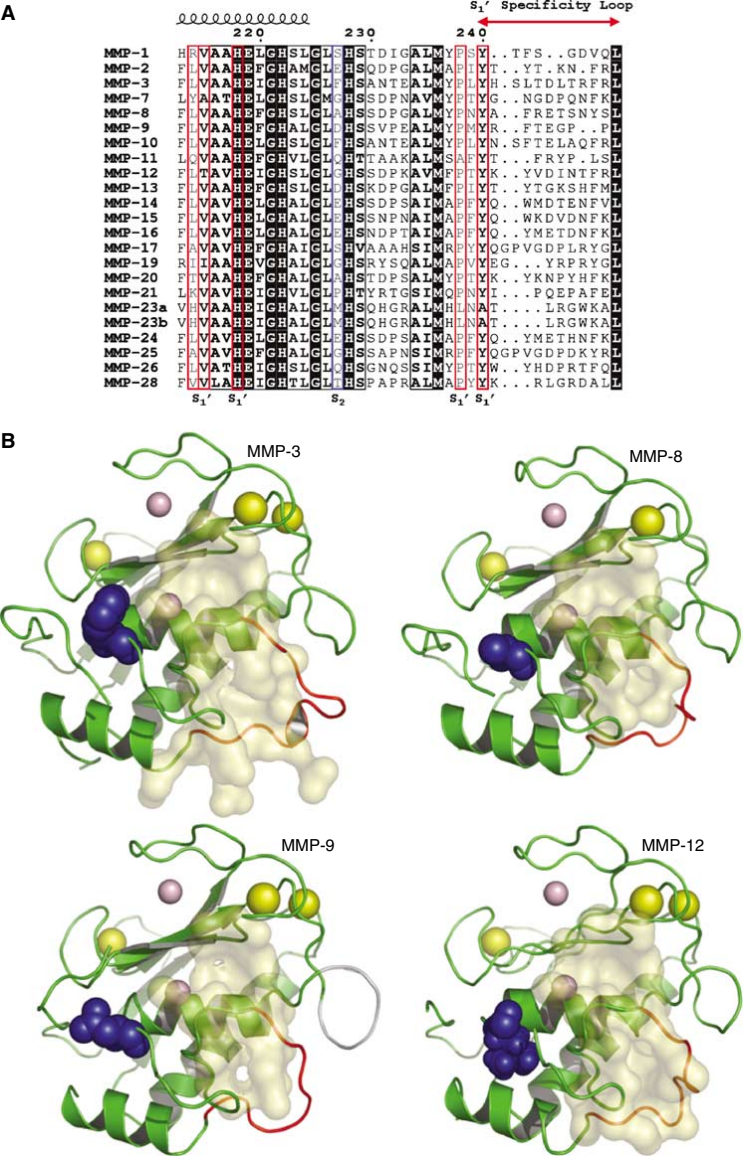
Sequence alignment of every human MMP active site. (**A**) The amino-acid sequences of MMP catalytic domain regions containing the S_1_′ subsite forming residues (red frame) and S_2_ essential residues (blue frame) were aligned using T-Coffee (Notredame C, Higgins DG, Heringa J (2000) T-Coffee: a novel method for fast and accurate multiple sequence alignment. *J Mol Biol* **302:** 205–217) and annotated using ESPrint (Gouet P, Courcelle E, Stuart DI, Metoz F (1999) ESPript: analysis of multiple sequence alignments in PostScript. *Bioinformatics* **15:** 305–308). The secondary structure and numbering is based on MMP1 Protein Data bank (PDB) #1HFC. (**B**) Structural representation of antitarget MMPs. The catalytic domains of MMP3 (PDB:1CIZ), MMP8 (PDB: 1KBC), MMP9 (PDB:1GKD) and MMP12 (PDB: 1Y93) were structurally aligned and superimposed. The empty voids of the catalytic pockets were calculated using CASTp (Liang J, Edelsbrunner H, Woodward C (1998) Anatomy of protein pockets and cavities: measurement of binding site geometry and implications for ligand design. *Protein Sci* **7:** 1884–1897) and visualised using Pymol (DeLano Scientific LLC, San Francisco, CA, USA http://www.pymol.org/) and its CATSp plugin. The S_1_′ pocket voids are in yellow, the essential S_2_ pocket residues at position 227 are shown in blue, and the S_1_′ specificity loop is shown in orange-red. The original structures contained a bound inhibitor in the active site, which was removed prior to calculation. Therefore, the S_1_′ voids include any structural adaptations in the molecule that were needed to accommodate the inhibitor. Although, these adaptations occurred upon binding of different inhibitors, the character of the void spaces is quite similar (data not shown).

The main sequence differences between MMP active sites reside in specificity loop residues that form the S_1_′ subsite pocket (Figure 1). This leads to structural and chemical differences in the S_1_′ subsite that are reflected in the substrate preferences of the shallow pocket MMPs (MMP1, 7) compared to deep pocket MMPs (2, 3, 8, 9, 12, 13). Nonetheless, most MMPIs lack specificity with only a few able to spare the shallow S_1_′ pocket MMPs by incorporating bulky or long side chains at P_1_′ (Overall and Kleifeld, 2006). Perhaps not surprisingly, the antitarget MMPs 3, -8, -9 and also -12, a potential antitarget (Overall and Kleifeld, 2006), have similar binding properties in the active site (Lukacova *et al*, 2004). Comparative modeling reveals that the void volumes of the MMP antitarget S_1_′pockets are very similar in size and shape (Figure 1B). This might be reflected by similarities in specificity towards key shared antitarget substrates involved in tumour protection. Indeed, the peptidic substrate preferences for MMP3 and MMP8 are very similar (Netzel-Arnett *et al*, 1991; Turk *et al*, 2001). So, MMPIs optimised to spare either of these MMP antitargets might spare the other.

The development of novel specific inhibitors for MMP12 (Dublanchet *et al*, 2005) and MMP13 (Chen *et al*, 2000) was attributed to differences in the S_1_′ pocket. An additional small region termed the ‘S_1_′ side-pocket’ or S_1_′^*^ was used for specifically targeting MMP13 (Chen *et al*, 2000). The success of these inhibitors clearly demonstrated the benefits of good mechanistic and structural information for inhibitor design. The MMP active site structure is generally rigid, but is very flexible in the S_1_′ pocket (Moy *et al*, 2002; Bertini *et al*, 2005; Cuniasse *et al*, 2005). This can be exploited to accommodate unfavorable binding groups in MMPIs. Indeed, the shallow S_1_′ pockets of MMP1 and MMP7 must be able to accommodate the bulky Phe and Tyr, respectively, for these are the P_1_′ residues cleaved during *in trans* zymogen autoactivation. However, active site flexibility renders SAR-based drug design challenging since it is difficult to predict the extent of molecular movement that can occur upon inhibitor binding. On the other hand, ‘shape shifter’ allosteric inhibitors that exploit active site flexibility to perturb subsite binding interactions or the catalytic centre are promising avenues for new MMPI development.

### Zinc-binding groups

**Figure 2:**
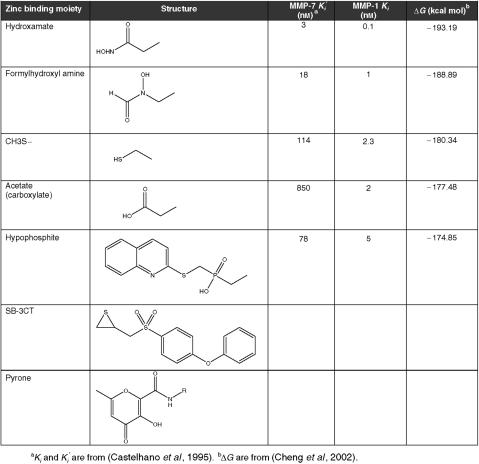
The structures of zinc binding groups, their inhibitory constants, and calculated free energy for interaction with MMP catalytic zinc. The lower free energy (Δ*G*) for interaction with MMP catalytic zinc of hydroxamate-based inhibitors is well correlated with their superior inhibitory constants relative to other common zinc-binding groups. This attribute of hydroxamate-based inhibitors conceals the contribution of other groups in the inhibitor structure, and therefore reduces their selectivity.

Zn^2+^-chelating hydroxamates have been favoured in MMPI design because of superior Δ*G* values, but a number of other groups are possible (Figure 2). However, strong Zn^2+^-chelating moieties disproportionately drive binding and so overwhelm the contribution from the rest of the compound, reducing other opportunities for improved specificity. Indeed, hydroxamate activity-based MMP probes related to marimastat bound many off-target metalloproteinases that were not MMPs (Saghatelian *et al*, 2004). This may be related to the lack of selectivity of hydroxamic acid for zinc over other divalent transition metals, including the ability to bind metal ions in several oxidation states such as iron (III). In addition, the hydroxamate carbon-nitrogen bond (C[=O]NHOH) can easily change to the *trans* configuration reducing its affinity (Puerta *et al*, 2004).

So, is there a way to utilise binding to the catalytic Zn^2+^ ion but also develop specificity? If weaker Zn^2+^-chelation groups were utilised, then by reducing the reliance on Zn^2+^-binding for potency, the chemistry would have to be optimised in other parts of the compound in order to still generate nanomolar inhibitors. To achieve the highest binding energies, the compound would have to be adapted for individual MMPs and so should lead to higher specificity MMPIs. Since TIMPs do not cause the musculoskeletal side effects that occurred with some MMPIs, then it is possible that neither MMPs nor ADAMs are the cause of these adverse reactions. This further supports incorporating weaker Zn^2+^ ion binding groups into the third generation MMPIs to reduce off target binding that was seen in activity based probe studies (Saghatelian *et al*, 2004). An alternative view though is that more potent and selective zinc-binding groups might improve MMPI specificity. For example very high affinity pyrone-based MMPIs (Figure 2) showed nanomolar potency and selectivity for MMP3 (Puerta *et al*, 2005).

### Active site prime side coordination

The nonprime side of the active site has not been used in inhibitors that reached clinical trials. By binding to both sides of the active site, potency should increase and new opportunities for specificity can be exploited. In some phosphinic inhibitors (Figure 2), the weak zinc chelating phosphinyl group was used on optimised backbones to generate extremely potent MMPIs that were two log orders selective for MMP11 (Cuniasse *et al*, 2005). These compounds mimic the transition state in peptide cleavage with the two phosphinic oxygens tetrahedrally orientated to the inhibitor backbone. This also allows for the inhibitor to be extended into the nonprime side, a feature that hydroxamate chemistry precludes. However, the negative charge renders this compound class membrane impermeable, limiting their distribution *in vivo*. Analysis of sequence alignments of the MMP active sites confirms the active site homology and reveals a variable residue at position 227 that defines the S_2_ pocket (Figure 1B). A number of MMPs have Glu or Asp here, whereas hydrophobic residues are found in other MMPs. By exploiting this acidic character, inhibitor selectivity may be engendered by charge attraction or repulsion.

### Covalent inhibitors

The fear of unanticipated side effects through accumulation of covalent-bound off targets motivates current drug development efforts for noncovalent inhibitor classes. However, this may be an unjustified strategy. Surprisingly, of the 317 currently licensed drugs in the USA that inhibit an enzyme target, 35% of the 71 enzyme targets are irreversibly blocked by mechanism-based compounds that covalently modify the target or enzyme–substrate complex (Robertson, 2005). These drugs have proven efficacy by targeting the catalytic mechanism rather than just focusing on binding. Since proteolytic enzymes hydrolyze peptide or isopeptide bonds, this catalytic mechanism can be exploited to develop unique drug classes. Upon binding, these suicide inhibitors or substrates undergo a conversion of an unreactive group in the inhibitor into a functionality reactive group that covalently modifies residues in the active site. Since the reactive species is formed only within the active site of the targeted enzyme, it provides high specificity and *in vivo* selectivity.

A potent mechanism-based thiirane sulphur-containing anti-MMP2 and -9 inhibitor that forms a reversible covalent bond with the active site glutamate (Figure 2), performs impressively in an aggressive murine model of T-cell lymphoma (Kruger *et al*, 2005). Recently, the design of the prototypic inhibitor was modified and a new generation of mechanism-based MMP2-specific MMPIs were developed (Ikejiri *et al*, 2005). So, although pharmaceutical companies would prefer to develop noncovalent inhibitors, for short or medium duration patient dosing or in very serious cancers, the risk of side effects may be acceptably low to consider the use of this class of compound.

### Exosite binding and allosteric inhibitors

Because the catalytic site and binding pockets of MMPs are structurally very similar, specificity might be also achieved with substrate-specific exosite inhibitors (Overall, 2002; Overall and López-Otin, 2002). Exosites on the hemopexin C domain of MMPs and collagen binding fibronectin type II modules in MMP2 and -9 drive catalysis of many substrates including chemokines and collagen (reviewed by Overall, 2002). The binding affinity of exosite domains for substrates is typically low (10^−6^–10^−7^ M) on generally featureless sites, rendering it potentially difficult to develop compounds that bind here. However, exosite binders may be linked to active site inhibitors to achieve highly potent and highly selective two-site binding inhibitors.

Moving away from ‘lock and key’ SAR principles for protease inhibitor design might lead to new allosteric, noncompetitive inhibitors that bind sites that are distant from the active and substrate-binding sites. Upon binding, a conformational change occurs in the target that reduces the catalytic rate. The individual rates of substrate binding and active site conformational change now become rate limiting in catalysis. Since the potency of competitive inhibitors is reduced as the concentration of substrate rises, allosteric noncompetitive or uncompetitive inhibitors that bind in the presence of substrates and maintain their efficacy have many advantages. Further, if allosteric sites are not shared by many MMPs, this approach represents a very promising approach for MMPI specifity.

Allosteric inhibitors might have been achieved for MMP12. A high throughput screen aimed to find non-zinc chelating compounds that showed additive inhibition against MMP12 over that achieved in the presence of a hydroxamate inhibitor alone (Dublanchet *et al*, 2005). Two nonpeptidic non-zinc chelating inhibitors in the S_1_′ pocket were found that could be crystallised in the presence of an acetohydroxamate anion that bound the active site Zn^2+^ ion (Morales *et al*, 2004). A novel subnanomolar nonchelating MMP13 inhibitor was also reported that binds deeply into the S_1_′ pocket of MMP13 and protrudes into the S_1_′^*^ side pocket. In this bent conformation, it clamps around a leucyl side chain, which is unique to MMP13 (Engel *et al*, 2005). However, it is not clear whether these are true allosteric inhibitors or functioning by competing for S_1_′ subsite occupancy in deep pocket MMPs. If such drug classes can be exploited for other MMPs, they may form an effective new strategy to specifically inhibit MMP drug targets.

## FUTURE PERSPECTIVES

A number of crucial issues must be resolved in order for effective treatments of cancer to be developed based on MMP inhibition. Matrix metalloproteinase candidate targets and antitargets must be fully validated and their biological roles in normal processes, host protection, and cancer must be clarified. In this task, systems biology analysis of the protease web – degradomics in its broadest sense – is the new frontier in proteolysis research and drug development. These approaches will be invaluable in leading us to new perspectives in understanding disease and in identifying new drug targets and antitargets. Exploiting a therapeutic opportunity is an exercise in risk management. So, when does treating a disease with a drug that unavoidably blocks some antitarget activities outweigh the tradeoff between benefit and harm? Even if only occurring rarely, the negative effects of antitarget blockade will eventually manifest when large populations are exposed, risking overall drug failure and posing human safety and litigation concerns for pharmaceutical companies. Hence, it is critical to develop rigid drug target validation criteria and to understand whether an antitarget is cancer type or even disease context dependent. Do the harmful effects of unintentionally blocking a cancer antitarget develop for all cancer types? Do the cancer antitarget effects that lead to accelerated carcinogenesis only occur when cancer is already present or might cancer be initiated in some individuals if a MMP is therapeutically inhibited in the treatment of other diseases? Hence, failure to develop innovative chemistry that spares cancer antitargets over MMP targets might render some MMPs as validated, but nondruggable cancer targets. Finally, we need to understand and avoid drug side effects through unexpected perturbations in the protease web resulting from therapeutic inhibition of key nodal MMPs that control the overall activity of other proteases in the degradome. Challenging? Yes. Is there therapeutic potential in decapitating MMP signaling cascades that promote tumorigenesis? Yes, but this remains unexplored and is therefore a bright light to replace the gloom following the MMPI clinical trials.
